# Effects of Deqi on Autonomic Balance in Adult Tinnitus Patients: Study Design of a Randomized Controlled Trial

**DOI:** 10.1155/2013/756012

**Published:** 2013-07-29

**Authors:** Qian-Qian Li, Guang-Xia Shi, Xin-Xing Fu, Li-Li Han, Li-Ying Liu, Cun-Zhi Liu, Lin-Peng Wang, Na Hou

**Affiliations:** ^1^Acupuncture and Moxibustion Center, Beijing Hospital of Traditional Chinese Medicine affiliated to Capital Medical University, 23 Meishuguanhou Street, Dongcheng District, Beijing 100010, China; ^2^Otorhinolaryngology, Institute of Beijing, Beijing 100730, China; ^3^Otolaryngology Department, Beijing Hospital of Traditional Chinese Medicine affiliated with Capital Medical University, 23 Meishuguanhou Street, Dongcheng District, Beijing 100010, China

## Abstract

*Background*. Recent reports suggest that a proportion of tinnitus patients suffer from mental illness. Autonomic nervous system plays a useful role in tinnitus therapy since electrical vagal nerve stimulation (VNS) has been frequently used to alleviate tinnitus-induced depression in clinic. heart rate variability (HRV), which is reflective of autonomic nervous system function, has been proved to be modulated by acupuncture. In the present study, we aim to compare the effect of deqi sensation on heart rate variability in adult tinnitus patients. *Methods*. Thirty participants are randomly assigned to verum acupuncture (creating deqi) or shallow acupuncture (not creating deqi) at Baihui (Du-20), Shenting (Du-24), Tinghui (GB-2), Waiguan (SJ-5), and Zulinqi (GB-41) for 3 weeks. The primary outcome measure is heart rate variability, which is measured at the first acupuncture, as well as the last acupuncture. *Discussion*. Completion of this trial will help to identify the role of deqi sensation in acupuncture effect for tinnitus and reveal an autonomic modulation mechanism for acupuncture effect. *Trial Registration*. This trial is registered with International Standard Randomised Controlled Trial Number ISRCTN58013563.

## 1. Background

Tinnitus is generally defined as a perception of sound in the absence of an external acoustic stimulus. Subjective tinnitus affects approximately more than 600 million individuals worldwide [1, 2]. It has a prevalence of roughly 12–15% in the adult population [3–5]. The amount of distress in people suffering from tinnitus can be evaluated by validated tinnitus questionnaires. The imbalance of autonomic system leads to further increases in the sensitivity of the auditory system and reinforcement of the attentional filters, which makes tinnitus loudness and awareness more severe. This, in turn, exacerbates the level of stress and so on, making the tinnitus progressively worse over time [6]. A number of clinical trials have demonstrated the presence of psychopathological disorders and high depressive scores in tinnitus patients [7, 8]. For this reason, there may be a causal relationship between the molecular bases of these disorders. As a the neurotransmitter of the parasympathetic nervous system, acetylcholine (ACh) is likely to play a role in plasticity at many sites in the auditory system [9]. Animal models of tinnitus demonstrate changes in cholinergic mechanisms in auditory nuclei [10]. The cholinergic signaling is involved in many physiological functions and in some disease states such as anxiety and depression [11]. It is suggested that the limbic and autonomous nervous systems are largely responsible for troublesome tinnitus [12]. These findings in the literature suggest an association among ANS, depression, and tinnitus. Heart rate variability (HRV), as a simple and noninvasive quantitative marker of autonomic function [13, 14], has the potential to evaluate the autonomic status of tinnitus patients. 

Acupuncture, as a particularly complementary and alternative medicine (CAM), has frequently been used to treat tinnitus [15, 16]. It can improve the symptoms of tinnitus by the activating of endogenous opioid mechanisms and neuropeptides which stimulate specific brain structures [17]. Acupuncture can relieve the loudness and disturbing quality tinnitus immediately, and significantly improve the quality of life, tension and sleep [18]. During the verum acupuncture, needles create the specific needle sensation called “deqi” (“aching,” “dull,” “heavy,” “numb,” “radiating,” “spreading” and “tingling”) at specific acupuncture points [19]. Deqi is suggested to be the main mechanisms producing effects from acupuncture [20], by generating a release of spinal and supraspinal beta-endorphins, proinflammatory neuropeptides and an increase in peripheral circulation [21]. However, there is lack of adequate experimental data to indicate the relationship between deqi sensation and modulation effect of acupuncture on ANS [22]. Therefore, more evidences are needed to prove the specific effect of acupuncture. The aim of our study is to evaluate the effect of deqi on HRV in tinnitus patients. 

## 2. Methods

We perform the study according to common guidelines for clinical trials (Declaration of Helsinki, International Conference on Harmonisation (ICH)/WHO Good Clinical Practice standards (GCP) including certification by an external audit). It will strictly follow the CONSORT statement to report high-quality study results. The trial protocol has been approved by the Research Ethical Committee of Beijing Hospital of Traditional Chinese Medicine affiliated to Capital Medical University (Ref: 201213). This trial was registered with ISRCTN at Current Controlled Trials (ISRCTN58013563). 

### 2.1. Recruitment

Patients will be recruited in acupuncture clinic, Beijing Hospital of Traditional Chinese Medicine affiliated to Capital Medical University with a target sample size of 30 subjects. The trial is executed from March 2012 to June 2013.

### 2.2. Inclusion Criteria

Patients who meet all of the following conditions will be considered for enrollment [23]:

1. typical conditions of unilateral or bilateral tinnitus,
2. aged 18–65, either sex,
3. tinnitus duration of more than 3 months,
4. not receiving any treatment last 1 month,
5. normal language and intelligence ability to answer and fill in the questionnaire,
6. better understanding of acupuncture, and good compliance to the research observation and evaluation,
7. written and informed consent.

### 2.3. Exclusion Criteria

1. Objectivity tinnitus (objective tinnitus is audible to the examining/auscultating physician),
2. acute or intermittent tinnitus, history of Meniere disease, or tinnitus induced by middle ear/inner ear/small pons angle tumor,
3. underlying disease or history: otitis media, tympanic membrane perforation, or eustachian tube function obstacle,
4. acoustic neuroma, intracranial damage or use of any ototoxic drugs,
5. severe dysfunction of heart, kidneys or liver,
6. the serious original disease of hematopoietic system or endocrine system,
7. serious aphasia, depression syndrome or mental disease.

### 2.4. Randomisation and Blinding

After participants complete a baseline evaluation, a research coordinator (Guang-Xia Shi) who is uninvolved with data collection randomly allocates them to either verum acupuncture group or shallow acupuncture group by using a computer-generated, blocked random-allocation sequence with a block size of 6. The random codes are only known by another researcher (Qian- Qian Li) who is not involved in treatment and statistical analysis.

The assignment is done in a single-blind manner, in which the patients are blinded to treatment assignment. In order to minimize the placebo effects, patients are informed in a manner suggesting that two different types of acupuncture treatment are compared. One type is traditional acupuncture, another is novel acupuncture, and the effect of both is uncertain. Similar strategies of informed consent have been used in most previous acupuncture trials [24]. HRV analysis and assessment of THI are performed by a researcher (Li-Li Han), who is blinded to patients' treatment respectively. During the intervention, acupuncturist and all the researcher personnel are segregated immediately after the treatment starts and are instructed not to exchange information with each other.

### 2.5. Interventions

**Figure 1:**
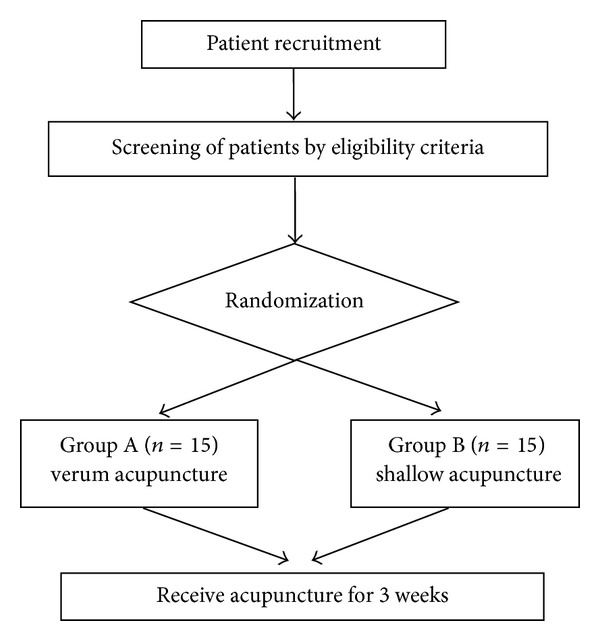
Flowchart.

This trial is a randomized controlled study. Participants will receive acupuncture for 3 weeks (Figure 1) (twice per week). All candidates go through a standardized interview and receive more information about the study and the treatments. They also undergo audiological testing of hearing thresholds, minimal masking levels, and loudness discomfort levels carried out by an audiologist in Tongren Hospital affiliated to Capital Medical University (Xin-Xin Fu).

Timepoints are as follow:

 visit 1: screening,
 visit 2: treatment initiation, participants will receive acupuncture for 3 weeks,
 visit 3: 3 weeks later of first acupuncture, followup and treatment finish.

Patients who meet the inclusion criteria and none of the exclusion criteria are randomized to one of two treatment groups.

 Group A will receive verum acupuncture (creating deqi sensation).
 Group B will receive shallow acupuncture (not creating deqi sensation).

**Figure 2:**
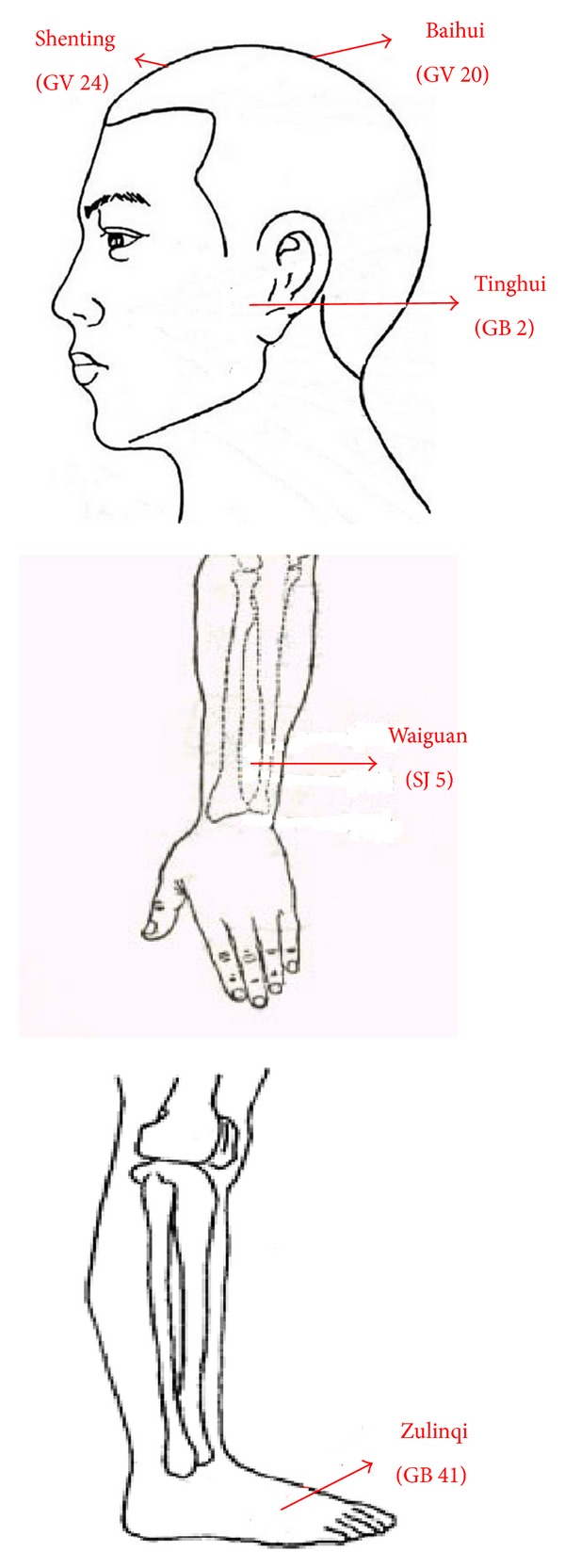
The points used in the trial.

Acupuncture points are selected as follow (Figure 2).

Patients are stimulated by 5 main acupoints: Baihui (Du-20), Shenting (Du-24), Tinghui (GB-2), Waiguan (SJ-5), and Zulinqi (GB-41). The acupoints are stimulated continuously by the true needles or the shallows for 20 minutes per session. 

Acupuncture is performed with sterile needles by one therapist (Cun-Zhi Liu) with more than 22 years of experience and an acupuncture license from the Chinese medicine practitioner from the Ministry of Health of China. Treatment will be conducted over a period of 3 weeks, at a frequency of 2 sessions/week. No additional treatment is allowed.

### 2.6. Primary Outcome Measures

**Figure 3:**
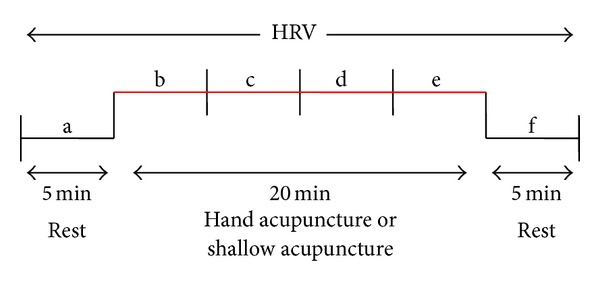
Sequence for heart rate variability (HRV) testing. The data before measurement phases (a), during (b–e), and after (f) were measured and statistically analyzed.

HRV indexes are measured using a digital 12 leads ECG-Holter machine (Mortara Instruments, USA). Within frequency domain, power in low frequencies (LF; 0.04–0.15 Hz), and in high frequencies (HF; 0.15–0.4 Hz) are calculated for each 5-minute density spectrum by integrating the power spectral density in the respective frequency bands. The HF/LF ratio also is analyzed [25]. HRV is measured at the first acupuncture, as well as the last acupuncture (Figure 3).

### 2.7. Secondary Outcome Measures

1. Change of tinnitus severity according to the tinnitus questionnaire of Tinnitus Handicap Inventory (THI) [26]:
  (1.1) F: functional subscale (11 factors),
  (1.2) E: emotional subscale (9 factors),
  (1.3) C: catastrophic subscale (5 factors).
2. Each question of the THI can be answered by the patient with either often (4 points), sometimes (2 point), or never (0 points) with a maximum total score of 100 indicating most severe suffering from tinnitus. The assessment is at baseline (before treatment initiation) and 3 weeks later of the first acupuncture.
3. Participants also report adverse events induced by the acupuncture treatment, including discomfort or bruising at the sites of needle insertion, nausea, or feeling faint after each treatment. Answers concerning possible side effects related to the acupuncture treatment will be documented by using a standardised questionnaire at the end of treatment.

### 2.8. Data Analysis

We use paired *t*-tests and two-way ANOVA with one repeated measure to analysis the data of 30 tinnitus patients. Post hoc analyze is performed using Tukey test. The level of significance is defined as *P* < 0.05. 

## 3. Discussion

This trial has been designed above to identify whether acupuncture could (verum acupuncture or shallow acupuncture) treat subjective tinnitus patients by modulating HRV, so as to explain the mechanism of acupuncture effects on tinnitus. If this questionnaire results provide evidence for the safety of acupuncture, more patients with these conditions may be encouraged to seek out acupuncture. 

The present study aimed to help to draw more attention in acupuncture's potential for treating patients with tinnitus by autonomic regulation. Although a climate where acupuncture might be assumed to be ineffective exists [27, 28], there is inadequate evidence on which to base a judgment on acupuncture's effectiveness for tinnitus. Well-designed randomized controlled trials which can provide the acupuncture effect mechanism for tinnitus by scientific approach are needed.

Verum acupuncture is administered by twirling and lifting the needles until deqi occurred. One of the greatest challenges in acupuncture research is selecting an appropriate sham acupuncture control. There are several existing sham acupuncture methods including shallow needling on acupoint, shallow needling on nonacupoint and ritualized mock acupuncture. To avoide the difference of acupoint selection, shallow needling on acupoint is used as a control group in this trials. For the shallow group, the point selection is the same as the verum acupuncture group. However, the depth of needles inserted vertically to the subcutaneous level should be rigidly controlled over no more than 3 mm. Any sensation (deqi) or needle manipulation is prohibited in this group. Because the needles are inserted at the same points, the patients cannot distinguish the shallow acupuncture from the real one on the vision. 

Placebo effects of acupuncture are actively studied in many articles, and sham acupuncture is a representative method for an acupuncture placebo tool [29, 30]. But there is a lack of consensus on the most valid approach to establishing placebo acupuncture. Therefore, important aspects of the placebo acupuncture control need to be considered. In this trial, we will evaluate the effect of deqi on HRV and reveal the potential mechanism of acupuncture treatment for tinnitus.

Needling sensation has been considered important to acupuncture therapeutic effects [31]. Deqi sensation is assumed by many acupuncturists to be associated with a therapeutic effect and is often sought during needling [32]. Based on a hierarchical cluster analysis, a grouping of seven sensations is found to be associated with the category of deqi (“aching,” “dull,” “heavy,” “numb,” “radiating,” “spreading,” and “tingling”) [19]. It has been determined that the changes in parasympathetic nervous activity were correlated with number of deqi sensations during acupuncture manipulation [33]. On the other hand, what degree does it affect the autonomic nerve is still unknown. It was concluded that at least a part of the effects of acupuncture was independent of the presence of a deqi sensation [34]. In this trial, we will compare the impact of needling at two different depths by HRV analysis and discuss the relationship of deqi phenomenon and acupuncture effect from the view of autonomic modulation.

Many patients mention that tinnitus has developed in a stressful life episode and that it is worsened by stressful situations [35]. In addition, tinnitus distress is related to autonomic changes in the sympathvagal balance [36]. However, there are few reports about the relationship between tinnitus and HRV, which was used as an objective noninvasive marker of the autonomic nervous system [37]. HRV monitoring is used to evaluate acupuncture-induced effects on the autonomic nervous system regulation [38]. In our study, the relations of HRV with tinnitus and HRV with acupuncture will be further discussed.

It should be acknowledged that our study has several potential limitations. First, the sample size is limited. Because there are few similar studies reported previously, we cannot calculate sample size on the basis of the differences of HRV modulated by verum acupuncture or shallow acupuncture in tinnitus patients. Second, shallow acupuncture may have some physiological effects, which is also a limitation of the study.
